# Morphological and molecular analyses of Anodontinae species (Bivalvia, Unionidae) of Lake Baikal and Transbaikalia

**DOI:** 10.1371/journal.pone.0194944

**Published:** 2018-04-09

**Authors:** Olga K. Klishko, Manuel Lopes-Lima, Arthur E. Bogan, Dmitry V. Matafonov, Elsa Froufe

**Affiliations:** 1 Institute of Natural Resources, Ecology and Cryology Siberian Branch, Russian Academy of Sciences, Chita, Russia; 2 CIBIO/InBIO—Research Centre in Biodiversity and Genetic Resources, University of Porto, Vairão, Portugal; 3 CIIMAR/CIMAR—Interdisciplinary Centre of Marine and Environmental Research, University of Porto, Matosinhos, Portugal; 4 SSC/IUCN—Mollusc Specialist Group, Species Survival Commission, International Union for Conservation of Nature, Cambridge, United Kingdom; 5 Research Laboratory, North Carolina State Museum of Natural Sciences, Raleigh, United States of America; 6 Institute of General and Experimental Biology Siberian Branch, Russian Academy of Sciences, Ulan-Ude, Russia; University of California, UNITED STATES

## Abstract

The diversity and taxonomy of anodontine species in Lake Baikal and Transbaikalia region has been contentious since it is based on a typological species concept, the so called “Comparatory Method”. Using this method, six Comparatory anodontine species have been described for the study area as belonging to the genus *Colletopterum*. This genus was separated from *Anodonta* based on shell characteristics and further split into two subgenera, i.e. *Colletopterum* sensu stricto and *Colletopterum* (*Piscinaliana)*. However, many authors do not recognize this separation maintaining all *Colletopterum* forms within *Anodonta*. The current study clarifies the taxonomy and systematics of Anodontinae in this region, using a combination of molecular, morphological and anatomical data. All previously recognized Comparatory forms are here recognized as a single species, i.e. *Anodonta anatina*.

## Introduction

It is well established that the high intraspecific phenotypic shell variability of freshwater mussels (Bivalvia: Unionida) is intrinsically connected with environmental variables [1], [2], [3], [4], [5], [6]. Additionally, other factors, such as sex and ontogeny may also influence shell shape [7], [8], [9], [10]. Due to these reasons, the use of shell shape is limited to identify taxonomic units in freshwater mussels. In fact, this has been a persistent and contentious problem that resulted in exaggerating the extant number of freshwater mussel species [11], [12]. The Russian taxonomy for freshwater bivalves, the Typological Species Concept with the Comparatory Method’ (TCS-CM) [13], [14], uses mainly the arc of maximal convexity of the valve’s outline (AMCVO) and the index of convexity (ratio of shell width to shell height) for species delimitation [15], [16]. This system is known to inflate the number of species, especially those with high morphological plasticity of shells, which only reflect different ecophenotypes. In fact, recent studies using a multi-disciplinary approach, combining genetic analyses with morphological and anatomical studies, significantly reduced the number of TCS-CM in different freshwater mussel taxa. For example, Bolotov et al. [17] significantly reduced the number of recognized TCS-CM margaritiferid species, and the same occurred in the Unionidae family with several works by Klishko et al. [18], [19], [20], [21].

The most comprehensive current global classification of freshwater mussels only recognizes three species of the genus *Anodonta* for Russia, i.e. *Anodonta anatina* Linnaeus 1758, *Anodonta cygnea* Linnaeus 1758, and *Anodonta beringiana* Middendorff, 1851 [22]. However, a recent study placed *A*. *beringiana* in the genus *Sinanodonta* [23]. While *A*. *anatina* is widespread from Western Europe to Lake Baikal, inhabiting a wide variety of habitats, *A*. *cygnea* occurs in a more restricted range (from Western Europe east to the Ural Mountains) and only in lentic habitats [24]. Both species present great phenotypic plasticity having several hundreds of synonyms assigned for each in their global distribution, including in Russia [11], [12], [22]. Moreover, synonyms of each species are often mixed and misidentified [25]. Recognizing that most of these names correspond to ecophenotypes, Zhadin [5] listed seven *Anodonta* species and [11] three, including *A*. *woodiana* and *A*. *beringiana* that are now excluded from the *Anodonta* genus [23].

European-Siberian anodontine bivalves were placed in the genus *Anodonta* until the first half of the twentieth century [5], [26], [27], [28]. Later they were divided into two genera–*Anodonta* and *Colletopterum* [29], [30], [31], [32], based on the differences of umbo sculpture, shell surface and size of adult shells. The generic name *Colletopterum* was established by Bourguignat [29] for specimens collected in the River Danube (Bulgaria), named as *C*. *praeclarum*. Using the inflated TCS-CM, genus *Colletopterum* was subsequently recognized in the identification keys of freshwater mollusks [15], [16], [33], [34]. Nine species in the genus *Colletopterum* were recorded for Russia by Starobogatov et al. [15]. Later, Bogatov et al. [16] recognize 12 species for Russia, splitting them in two subgenera *Colletopterum* s. str. and *Colletopterum* (*Piscinaliana*) (Table 1; [16]). Recently, the species that were retained within *Anodonta* in these keys were synonymized with *Anodonta cygnea* and all species of *Colletopterum* (*s*.*s*.) and *Colletopterum* (*P*.) in the same keys were synonymized under *Anodonta anatina* [12], [22].

**Table 1:** Classification of the species corresponding to the TCS-CM genus *Colletopterum* Bourguignat, 1880.

Zhadin, 1938	Starobogatov et al., 2004	Bogatov et al., 2005	This Study
**Genus *Anodonta*** Lamark, 1799	**Genus *Colletopterum*** Bourguignat, 1880	**Genus *Colletopterum***	**Genus *Anodonta***
**Subgenus** *Anodonta* s. str.		**Subgenus *Colletopterum*** s. str.	
**Species**	**Species**	**Species**	**Species**
***A*. *anatina*** Linnaeus, 1758**	***C*. *anatinum*** (Linnaeus, 1758)**	***C*. *apollonicum*** (Bourguignat, 1880)*	***A*. *anatina*** Linnaeus, 1758**
***A*. *piscinalis*** Nilsson, 1822**	***C*. *apollonicum*** (Bourguignat, 1880)*	***C*. *baeri*** Bogatov, Starobogatov & Prozorova, 2005*	
***A*. *sedakovi*** Siemaschko, 1848******	***C*. *depressum*** (Bourguignat, 1881)*	***C*. *convexum*** (Drouët 1888)*	
	***C*. *nilssonii*** (Küster, 1842)**	***C*. *milaschevichi*** Bogatov, Starobogatov & Prozorova, 2005*	
	***C*. *ponderosum*** (Pfeiffer, 1825)**	***C*. *ostiarium*** (Drouët 1881)*	
	***C*. *piscinale*** (Nilsson, 1822)**	***C*. *subcirculare*** (Clessin, 1873)*	
	***C*. *rostratum*** (Rossmaessler, 1836)**		
	***C*. *sorensianum*** (Dybowski, 1913)***	**Subgenus *Piscinaliana*** Bourguignat, 1880	
	***C*. *subcirculare*** (Clessin, 1873)*	**Species**	
		***C*. *anatinum*** (Linnaeus, 1758)**	
		***C*. *nilssonii*** (Küster, 1842)**	
		***C*. *piscinale*** (Nilsson, 1822)**	
		***C*. *ponderosum*** (Pfeiffer, 1825)**	
		***C*. *rostratum*** (Rossmaessler, 1836 **	
		***C*. *sorensianum*** (Dybowski, 1913)***	

Distribution:

* Europe, European Russia

** European Russia, Siberia to Baikal Lake and Transbaikalia

*** Baikal Lake, Transbaikalia.

Because the Russian identification keys were based mainly in conchological and geographical factors, a combined methodology using genetic analyses with morphological and anatomical studies is needed to clarify the classification of Russian anodontine species assigned to *Anodonta* and *Colletopterum* genera.

Lake Baikal is the oldest and deepest lake in the world and is known by its high biodiversity and endemism [35]. However, the recorded diversity for freshwater mussels is extremely poor. In fact, up to the late 1990s, only a single anodontine species was recognized in Lake Baikal, i.e. *Colletopterum* (*Ponderosiana*) *ponderosum sedakovi* [32]. This number has been recently increased to five species [36] using the TCS-CM contentious methodology.

The aim of the present study was to revise the systematics and taxonomy of Anodontinae species from Lake Baikal and adjacent territory of Transbaikalia, using an integrative morphological, anatomical, and molecular approach. Furthermore, the study also aims to compare these findings with the classification used by TCS-CM, for anodontines from the study area and for those with a European distribution.

## Materials and methods

### Study area and sampling

**Fig 1:**
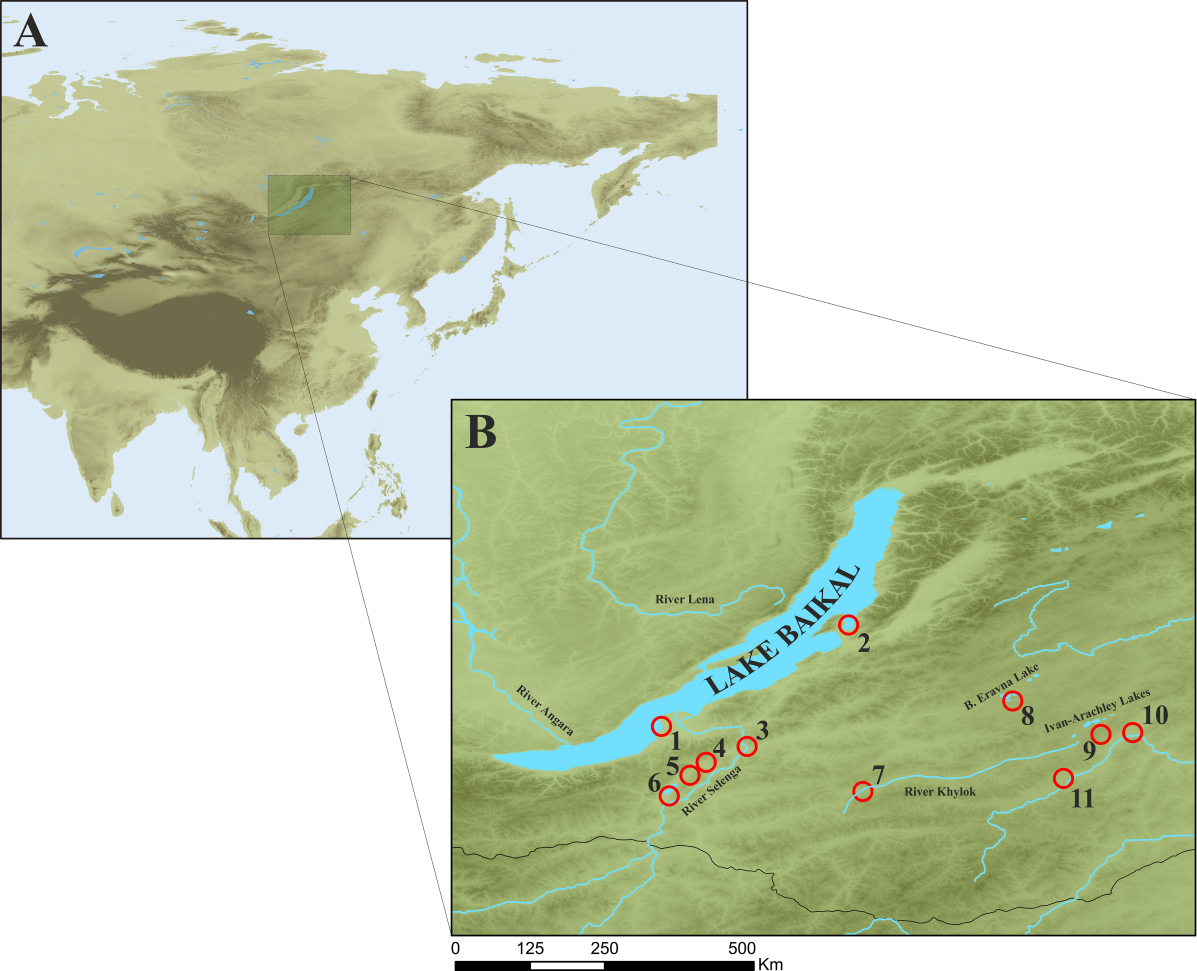
Map of the study area with anodontine specimens sampling sites (red circles). A–location of Lake Baikal in Russia territory, B–Map of Lake Baikal and Transbaikalia region; Lake Baikal: 1 –Cherkalov Sor and 2 –Chyvyrkuy Bay; Baikal Basin (3 –River Selenga, 4 –Lake Torma, 5 –Lake Schuchje, 6 –Gusinoye, 7 –River Khilok); River Lena basin (8 –Lake Bol’shoe Eravnoe); Baikal-Lena basin (9 –Ivan-Arachley lake system including Lakes Shaksha, Arachley, Kergendu, Ivan and Tasey), closed lake-refuges (10 –Lake Kenon, 11 –Lake Arey).

A total of 139 anodontine specimens were collected from Lake Baikal, Lakes Ivan-Arachley, and River Lena basins, Russia, by scuba diving and seine in the coastal areas, during the summer of 2004 and autumn of 2016. (Fig 1). All specimens were measured to the nearest 0.1 mm including shell length (L), shell width (B), maximal shell height (H_m_), and distance from umbo apex to the anterior shell margin (l). Additionally, the dimensions and shell characteristics of 29 specimens from European Russia were extracted from Zhadin [5] and Bogatov & Kijashko [33] for morphological and statistical analyses. Since freshwater mussels (*Colletopterum* sp.) are not rare, endangered or protected species, no permits are required for the collection of these mussels, in the study region. Additionally, field work did not involve any territory inside national parks or other protected areas. The map of the study area and collection site maps were built using QGIS 3.0 (Fig 1).

### Morphometry

**Fig 2:**
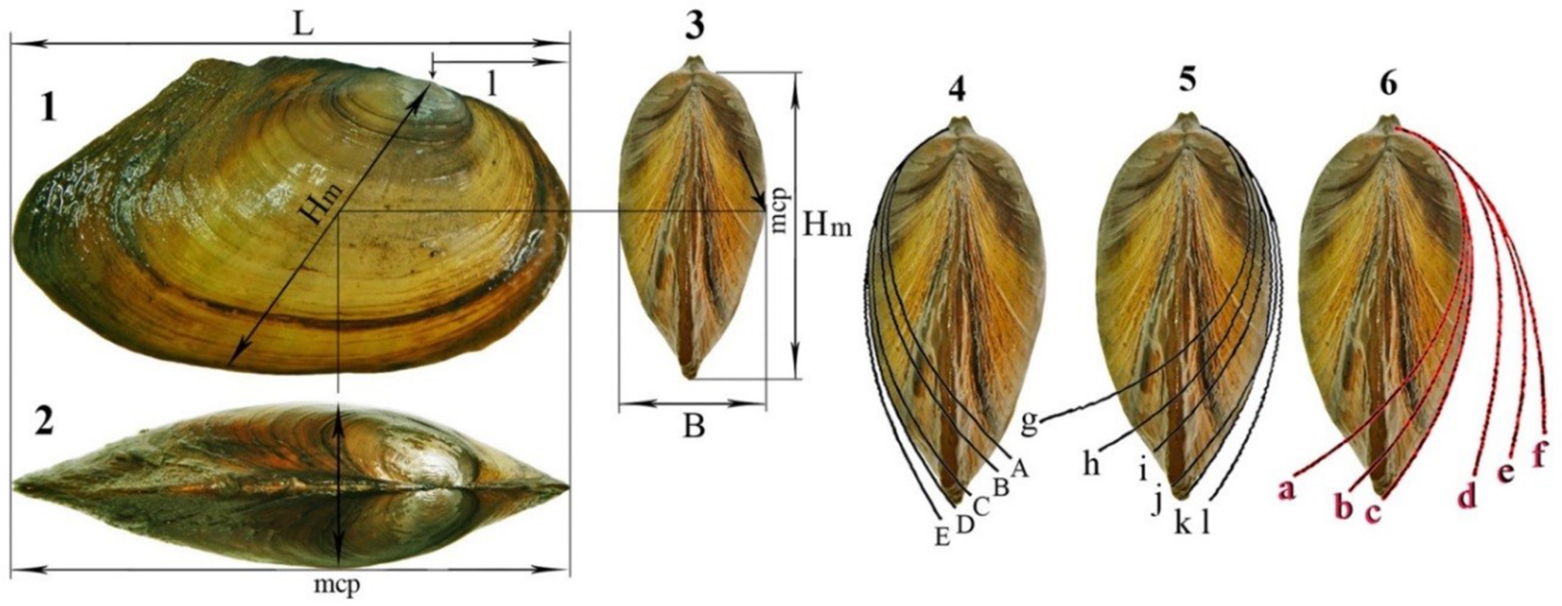
Shell measurements used for morphometry and species identification. **1 –**lateral shell view showing: the distance from umbo apex to anterior shell margin (**l**) and shell maximal height (**Hm**) from umbo apex to ventral shell margin across maximal convex point of valves (**mcp**); **2** –dorsal shell view showing the **mcp**; **3** –anterior shell view and shell width (**B**) in the maximal convex point (**mcp**); **4** –arcs of maximal convexity of the valve outline of Baikalian species (Prozorova & Bogatov, 2006): **A**–*C*. *ponderosum* Pfeiffer 1825, **B–***C*. *anatinum* Linnaeus 1758, **C**–*C*. *piscinale* Nilsson 1822, **D**–*C*. *nilssonii* Küester 1842, **E**–*C*. *sorensianum* Dybowsky, 1913; **5** –arcs of maximal convexity of the valve outline of Eurasian species: **g*–*** *С*. *rostratum* Rossmaessler 1836, **h*–****C*. *ponderosum*, **i*–****C*. *anatinum*, **j*–*** *С*. *piscinale*, **k*–****C*. *nilssonii*, **l*–****C*. *sorensianum*, and **6 –**of European species: **a**–*C*. *apollonicum* (Bourguignat 1880), **b**–*C*. *convexum* (Drouet 1888), **c**–*C*. *milaschevichi* Bogatov, Starobogatov et Prozorova 2005, **d**–*C*. *ostiarium* (Drouet 1881), **e**–*C*. *baeri* Bogatov, Starobogatov et Prozorova 2005, **f–***C*. *subcirculare* (Clessin 1873) according to Bogatov et al. (2005).

Standard morphometric shell characters, i.e. B/H, H/L and l/L ratios were calculated according to Bogatov et al. [16] (Panels 1–3 in Fig 2). TCS-CM species identification was based on shell shape, B/H and l/L indexes, and standard curves of the Arc of Maximal Convexity of the Valve’s Outline (AMCVO) [16], [36] (Panels 4–6 in Fig 2). The age of each specimen was estimated by counting annual growth rings. Statistical analysis was performed using Microsoft Excel, 2010.

### DNA extraction, PCR, sequencing and species identification

A total of 24 specimens collected from the study area, including at least 2 individuals per site and all previously identified TCS-CM species, were sampled for genetic analyses (Table 2). For comparison, six specimens from Ukraine were also included (Table 2). In detail, a small piece of foot tissue was clipped and stored individually in 96% ethanol. DNA extraction, PCR and sequencing conditions of F-type mtDNA cytochrome c oxidase subunit I (COI) followed Klishko et al. [20] (Annealing temperature 48-50ºC). Sequences were assembled using ChromasPro 1.7.4 (Technelysium, Tewantin, Australia). Alignment was performed in Bioedit 7.2.5 [37] including all newly sequenced individuals and all sequences from the most recent and comprehensive COI dataset for *Anodonta anatina* from Froufe et al. [25], with a total of 125 COI sequences. A haplotype network was constructed using TCS 1.21 [38] with a threshold of 95% and the output file was run through tcsBU [39]. Sequence divergences within the European haplogroup (uncorrected *p*-distance) were calculated using MEGA 7.0 [40]. Two distinct tools were used for this COI dataset to detect the number of Molecular Operational Taxonomic Units (MOTUs), and therefore the number of potential species. First, the Cluster Sequences tool implemented in BOLD 4 [41] was used and the generated BINs recognized as MOTUs. For the second, the 95% statistical parsimony connection limit using TCS 1.21 [38], was also applied.

**Table 2:** List of all individual specimens used for genetic analyses, collection sites and GenBank accession codes.

Species	Locality	Country	Code/GenBank	Comparatory species
*Anodonta anatina*	Lake Gusynoye	Russia	BIV3374/MH062766	*Colletopterum anatinum*
*Anodonta anatina*	Lake Gusynoye	Russia	BIV3375/MH062767	*Colletopterum ponderosum*
*Anodonta anatina*	Lake Schuchje	Russia	BIV3377/MH062768	*Colletopterum nilssonii*
*Anodonta anatina*	Lake Schuchje	Russia	BIV3378/MH062769	*Colletopterum piscinale*
*Anodonta anatina*	Lake Schuchje	Russia	BIV3379/MH062770	*Colletopterum anatinum*
*Anodonta anatina*	Lake Schuchje	Russia	BIV3381/MH062771	*Colletopterum ponderosum*
*Anodonta anatina*	Lake Torma	Russia	BIV3382/MH062772	*Colletopterum anatinum*
*Anodonta anatina*	Lake Torma	Russia	BIV3383/MH062773	*Colletopterum nilssonii*
*Anodonta anatina*	Lake Torma	Russia	BIV3384/MH062774	*Colletopterum ponderosum*
*Anodonta anatina*	Lake Torma	Russia	BIV3385/MH062775	*Colletopterum piscinale*
*Anodonta anatina*	River Selenga	Russia	BIV3387/MH062776	*Colletopterum sorensianum*
*Anodonta anatina*	River Selenga	Russia	BIV3388/MH062777	*Colletopterum sorensianum*
*Anodonta anatina*	Lake Big Eravna	Russia	BIV3392/MH062778	*Colletopterum sorensianum*
*Anodonta anatina*	Lake Big Eravna	Russia	BIV3393/MH062779	*Colletopterum nilssonii*
*Anodonta anatina*	Lake Big Eravna	Russia	BIV3394/MH062780	*Colletopterum sorensianum*
*Anodonta anatina*	Lake Big Eravna	Russia	BIV3395/MH062781	*Colletopterum piscinale*
*Anodonta anatina*	Lake Big Eravna	Russia	BIV3397/MH062782	*Colletopterum anatinum*
*Anodonta anatina*	Lake Big Eravna	Russia	BIV3399/MH062783	*Colletopterum ponderosum*
*Anodonta anatina*	Lake Baikal	Russia	BIV3401/MH062784	*Colletopterum rostratum*
*Anodonta anatina*	Lake Baikal	Russia	BIV3402/MH062785	*Colletopterum ponderosum*
*Anodonta anatina*	Lake Baikal	Russia	BIV3405/MH062786	*Colletopterum anatinum*
*Anodonta anatina*	Lake Baikal	Russia	BIV3406/MH062787	*Colletopterum sorensianum*
*Anodonta anatina*	Lake Baikal	Russia	BIV3408/MH062788	*Colletopterum piscinale*
*Anodonta anatina*	Lake Tasey	Russia	BIV0256/MH062765	*Colletopterum ponderosum*
*Anodonta anatina*	River Murafa	Ukraine	BIV0201/MH062760	*Colletopterum ponderosum*
*Anodonta anatina*	River Revna	Ukraine	BIV0202/MH062761	*Colletopterum ponderosum*
*Anodonta anatina*	River Revna	Ukraine	BIV0206/MH062764	*Colletopterum subcirculare*
*Anodonta anatina*	Pond in Dinotzky	Ukraine	BIV0204/MH062762	*Colletopterum piscinale*
*Anodonta anatina*	River Suchoy Tashlyk	Ukraine	BIV0205/MH062763	*Colletopterum subcirculare*
*Anodonta anatina*	River Vorsakla	Ukraine	BIV0199/MH062759	*Colletopterum minimum*

## Results

### General morphological features

Shell shape of collected specimens is highly variable, from ovate-rectangular and ovate-quadrangular to ovate-elongated with a curved or straight noticeable wing, on the dorsal margin (Table 3; S1–S6 Figs). Shell convexity varied from flattened to strongly convex. Periostracum is usually yellow-brown or green-yellow-brown; the nacre is white, yellowish or light blue. The anterior muscle scar is well imprinted and the posterior adductor scar is weakly visible, pseudocardinal and lateral teeth are absent.

**Table 3:** Shell metrics and TCS-CM identification of collected specimens.

Identification	Shell length	Shell width	Shell shape	B/H	H/L	l/L	AMCVO	TCS-CM
geography + TCS-CM	(mm)	(mm)						identification
***C*. *sorensianum***	28–80	1.5–1.9	ovate-quadrangular	0.35–0.44	0.64–0.82	0.19–0.25	*l*	*C*. *sorensianum*
			with wing				*e*	*C*. *baeri*
							*f*	*C*. *subcirculare*
***C*. *nilssonii***	48–87	1.6–2.2	ovate-quadrangular	0.41–0.47	0.55–0.77	0.21–0.27	*k*	*C*. *nilssonii*
			wing is low				*c*	*C*. *milaschevichi*
							*d*	*C*. *ostiarium*
***C*. *piscinale***	61–96	1.7–2.5	ovate-quadrangular or elongated	0.52–0.57	0.54–0.67	0.21–0.33	*j*	*C*. *piscinale*
			wing is low				*b*	*C*. *convexum*
***C*. *anatinum***	61–112	2.2–2.7	ovate-rectangular or quadrangular	0.52–0.57	0.52–0.62	0.21–0.33	*i*	*C*. *anatinum*
			wing almost unnoticeable				*a*	*C*. *apollonicum*
							*b*	*C*. *convexum*
							*c*	*C*. *milaschevichi*
							*h*	*C*. *ponderosum*
***C*. *ponderosum***	68–112	2.5–2.8	ovate-rectangular or elongated	0.58–0.63	0.52–0.63	0.18–0.27	*h*	*C*. *ponderosum*
			without wing				*a*	*C*. *apollonicum*
							*b*	*C*. *convexum*
							*i*	*C*. *anatinum*
***C*. *rostratum***	79–125	2.8–3.1	elongated oval, almost cylindrical	0.66–0.75	0.45–0.58	0.17–0.23	*g*	*C*. *rostratum*
			without wing				*a*	*C*. *apollonicum*

**Fig 3:**
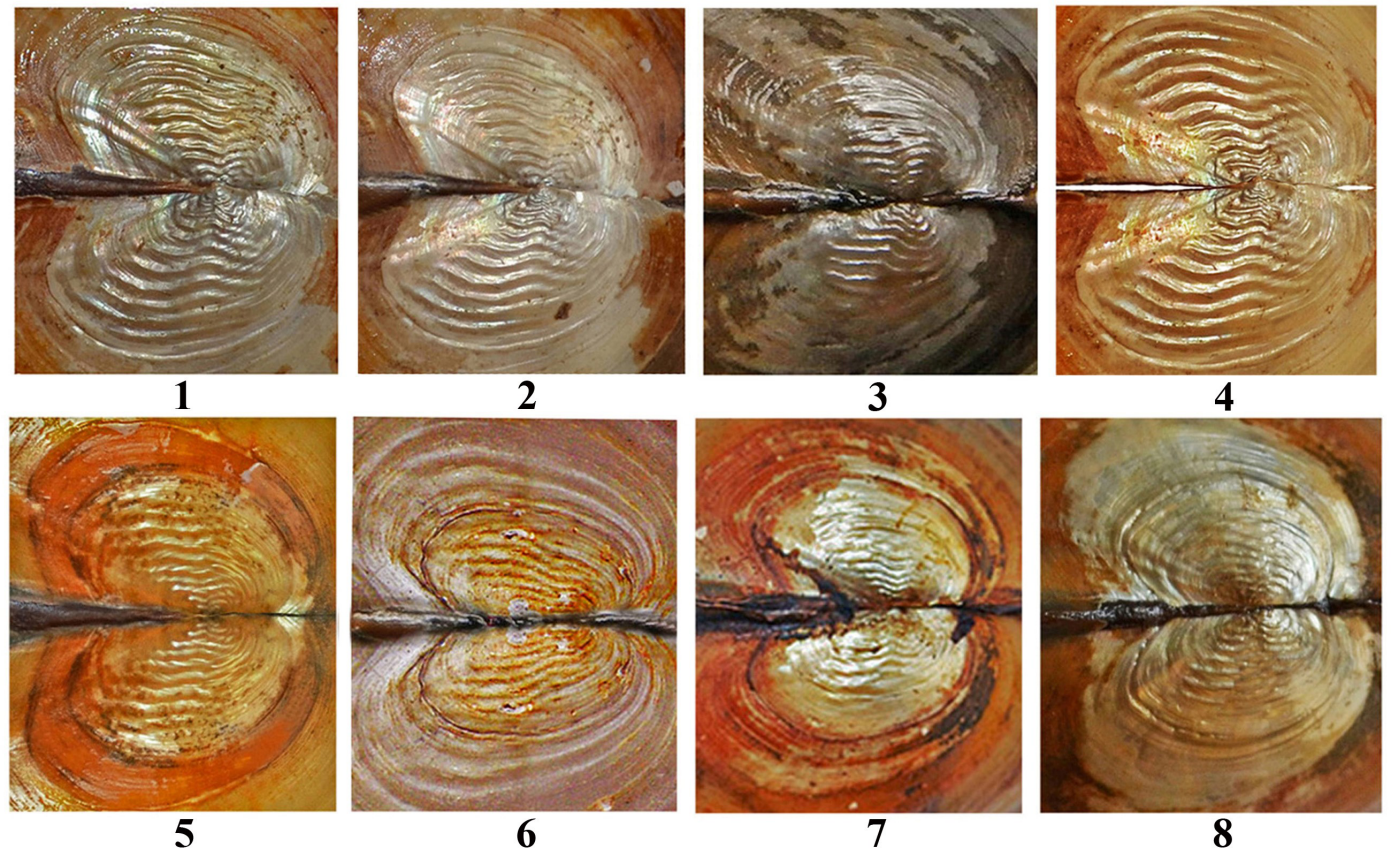
Umbo sculpture in *Colletopterum* species from studied region of Lake Baikal and Transbaikalia.

The umbo is wide and often eroded and does not project above the dorsal shell margin, its position from the anterior shell margin varies from 0.18 to 0.36% of the total shell length. Umbo sculpture of all examined specimens is double-looped [42] (Panels 1–4 in Fig 3) or with almost straight bars (Panels 5 and 6 in Fig 3), sometimes with discrete lines (Panels 7 and 8 in Fig 3). Umbo sculpture is strongly pronounced in some animals but weakly marked in others, regardless of age.

**Fig 4:**
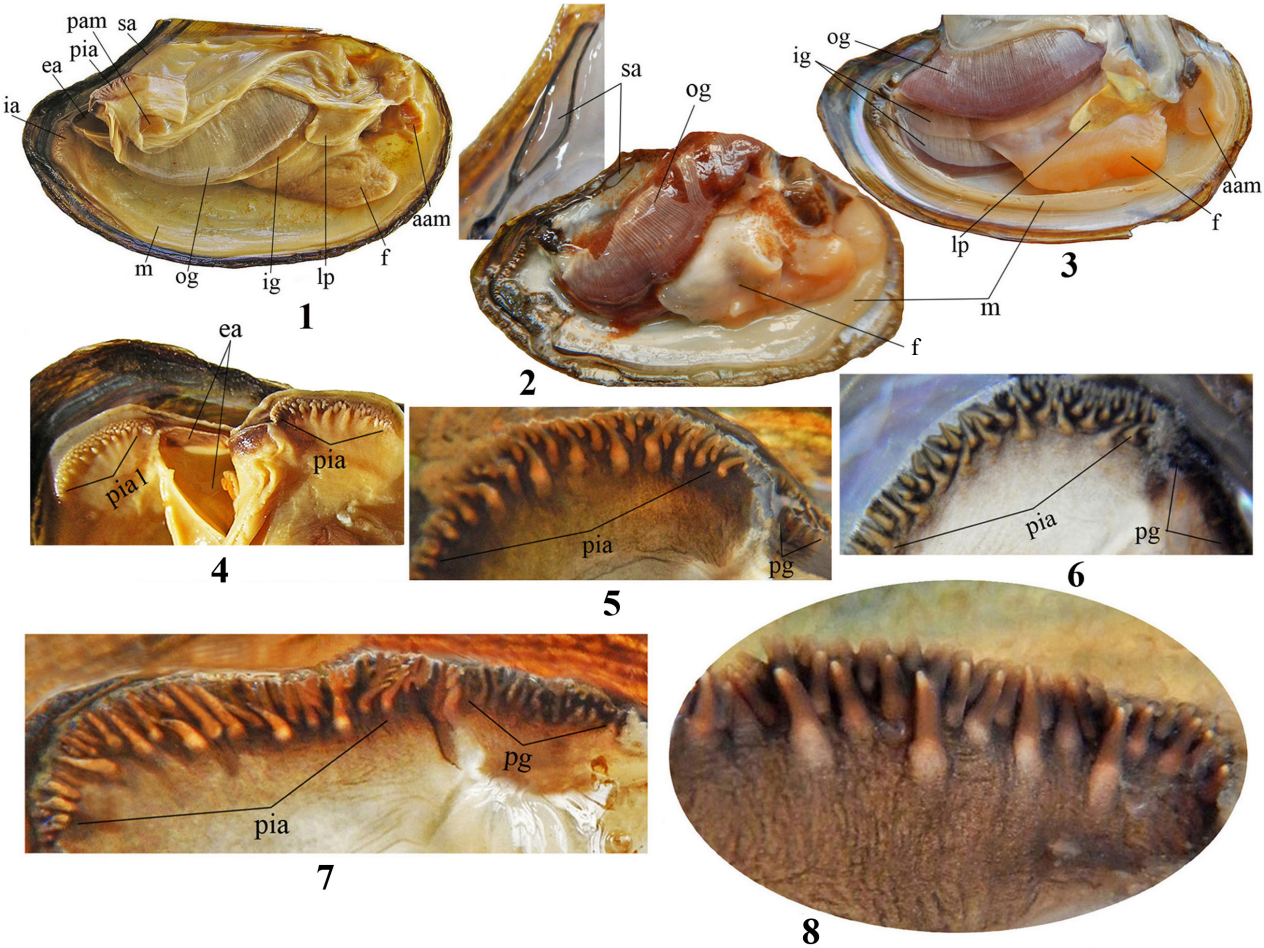
Inner anatomy features in *Colletopterum* specimens from Lake Baikal and Transbaikalia. General view of soft body **4.1 –**male, **4.2–4.3** –female with mature glochidia: **m**–mantle, **ig**–inner gills, **og**–outer gills, **lp**–labial palps, **f**–foot, **ia**–inner aperture, **ea**–excurrent aperture, **sa**–supra-anal aperture, **aam**–anterior adductor muscle, **pam**–posterior adductor muscle; **4.4**: **pia1 –**retracted position and **pia**–normal position of papillae in the inner aperture, **4.5–4.8**: **pia**–papillae shape of the inner aperture and **pg**–different pigmentation of excurrent aperture, **4.9 –**papillae scaled-up.

Soft body anatomy (or internal anatomy) is not dependent of shell shape and is similar in all specimens from the Lake Baikal, Baikal drainage and outside (Panels 1–3 in Fig 4). The mantle is an even cream color (Panels 1–3 in Fig 4: m). The inner and outer gills have the same shape and size, but the outer gills in gravid female have greater size than inner ones. Glochidia develop in the outer gills of female (Panels 2 and 3 in Fig 4.: ig, og). The foot is cream colored being darker or orange in the ventral part. The labial palps are well developed and tongue shaped (Panels 1–3 in Fig 4: f, lp). There are three mantle apertures: incurrent, excurrent and supra-anal (Panels 1 and 2 in Fig 4: ia, ea and sa). The papillae on the incurrent aperture are well developed, elongate-conical with swelling in base, with inner surface flat and convex on the outside. Sometimes papillae can be retracted due to preservation (Panels 4–9 in Fig 4: pia, pia1). The excurrent aperture lacks any papillae and has an unvarying pigmentation or pigmentation imitating papillae (Panels 6–8 in Fig 4: pg).

### Morphometry and species identification

Based on the TCS-CM and geographic distribution, six Comparatory species of *Colletopterum* (*Piscinaliana*) were identified in the study region: *C*. *sorensianum*, *C*. *nilssonii*, *C*. *piscinale*, *C*. *anatinum*, *C*. *ponderosum* and *C*. *rostratum* (Table 4, S1–S6 Figs). However, based only on the TCS-CM identification, i.e. the standard curves of the arc of maximal convexity of the valve’s outline (AMCVO) and morphometric indices, each separate TCS-CM species of *Colletopterum* (*P*.) from the studied region may also be identified as other TCS-CM species of *Colletopterum* s. str. from the European distribution (Table 3).

**Table 4:** TCS-CM species distribution of collected specimens. Presence +, Absence -.

TCS-CM species	Sampling sites													
	Baikal Lake		Baikal Lake Basin					Lena River Basin	Baikal Lake-Lena River Basin				Closed lakes	
	1	2	3	4	5	6	7	8	9	10	11	12	13	14
*C*. *sorensianum*	+	+	+	-	-	-	-	+	-	+	-	-	-	-
*C*. *nilssonii*	+	+	-	+	+	+	-	+	+	+	+	-	-	-
*C*. *piscinale*	+	+	-	+	+	-	-	+	+	+	-	+	-	-
*C*. *anatinum*	+	+	+	+	+	+	+	+	+	+	-	+	-	+
*C*. *ponderosum*	+	+	-	+	+	+	-	+	+	+	+	-	-	-
*C*. *rostratum*	+	-	-	-	-	+	-	-	-	-	-	-	+	-

1 –Cherkalov Sor, 2 –Chyvyrkuy Bay, 3 –Selenga River, 4—Torma Lake, 5 –Shuchyje Lake, 6 –Gusinoe Lake, 7 –Khylok River, 8 –Bol’shoe Eravnoe Lake, 9–12 –Ivan-Arachley lakes system (including: 9 –Arachley Lake, 10 –Kergendu Lake, 11 –Tasey Lake, 12 –Ivan Lake), 13 –Kenon Lake and 14 –Arey Lake.

### Morphometric analyses

**Fig 5:**
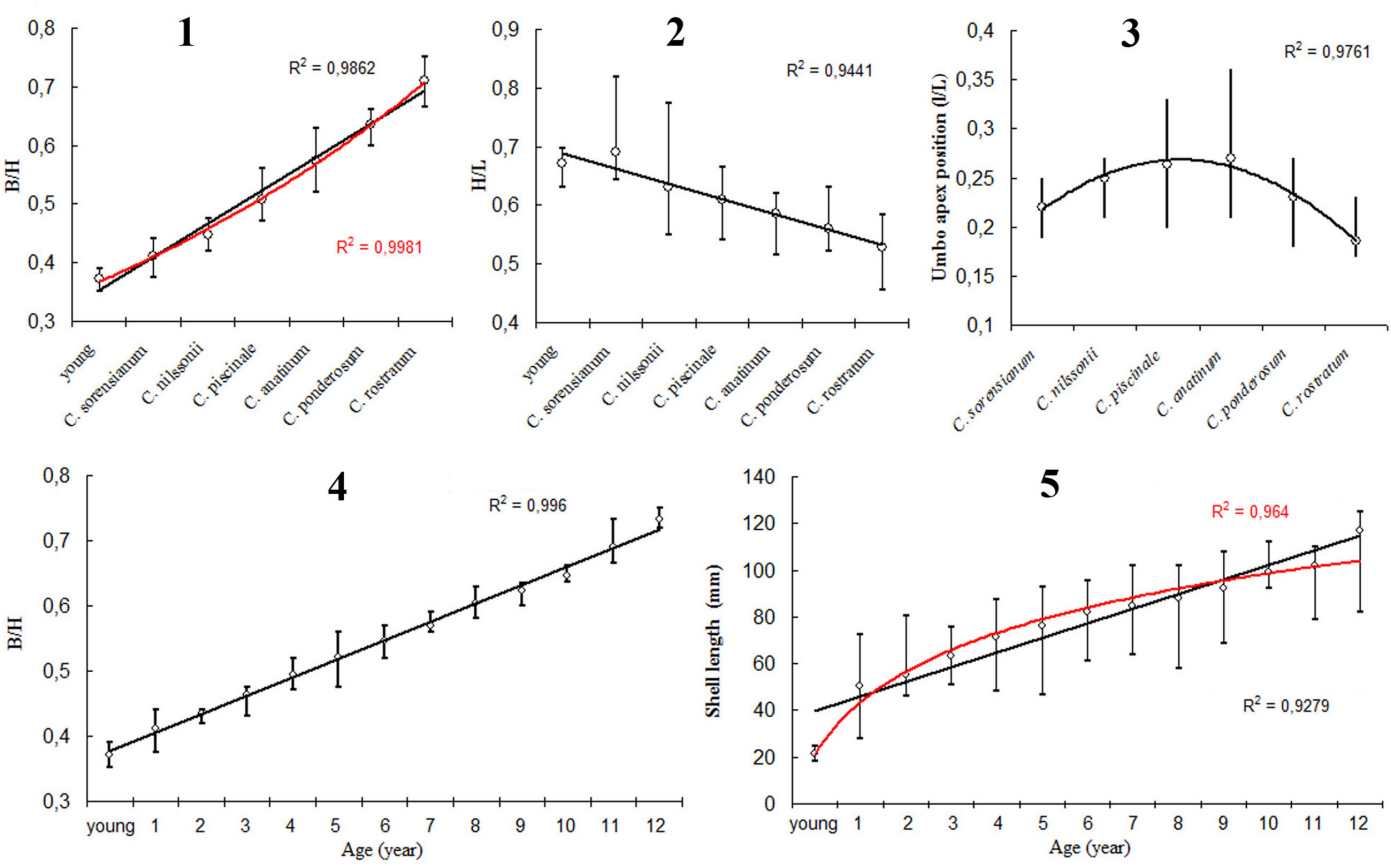
Intraspecific variability and dynamic of mean values of morphometric shell indexes. B/H– **5.1**, H/L– **5.2**, and l/L– **5.3** in *Colletopterum* species, range and change of mean values of B/H– **5.4** and shell length– **5.5** with increase of animal age from different localities in Lake Baikal, Transbaikalia and European Russia.

**Fig 6:**
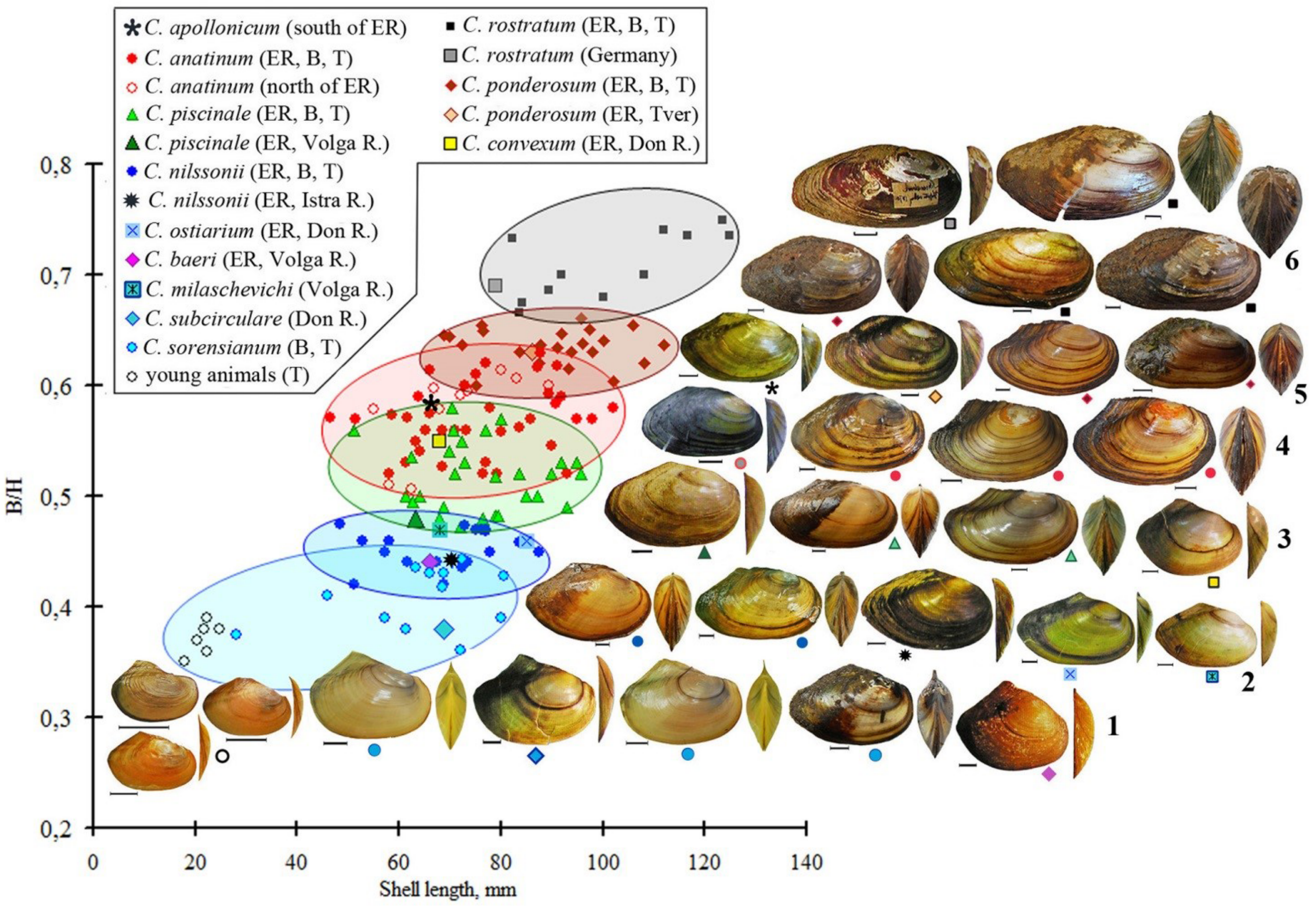
Change of shell shape and shell convexity with growth of shell length and index B/H in different age groups of TCS-CM *Colletopterum* species from different localities. **B**–Lake Baikal, **T**–Transbaikalia, **ER**–European Russia: **1** –young and group of *C*. *sorensianum* including *C*. *subcirculare* and *C*. *baeri*, **2** –group of *C*. *nilssonii* including *C*. *ostiarium* and *C*. *milaschevichi*, **3 –**group of *C*. *piscinale* including *C*. *convexum*, **4 –**group of *C*. *anatinum* with transitional form *C*. *apollonicum*, **5** –group of *C*. *ponderosum* and **6** –group of *С*. *rostratum*.

Analyses of morphometric shell indexes B/H, H/L and l/L have shown significant variability and overlap in *Colletopterum* species from different localities of Lake Baikal, Transbaikalia and European Russia. This overlap can be substantial, even almost complete, in the TCS-CM species *C*. *anatinum* and *C*. *piscinale* (Table 3). The lower values of B/H were characteristic for all young individuals from all species and for *C*. *sorensianum* (including *C*. *subcirculare* and *C*. *baeri*). These values increased in *C*. *nilssonii* (including *C*. *milaschewichi* and *C*. *ostiarium*) and *C*. *piscinale* (including *C*. *covexum*) (Table 3; Panel 1 in Fig 5), with the higher values being obtained in *C*. *ponderosum* and *C*. *rostratum* (Table 3; Panel 1 in Fig 5). The values of H/L decrease in the reverse order (Panel 2 in Fig 5). Following the same order from *C*. *sorensianum* to *C*. *rostratum*, the position of the umbo (l/H) in the same species increases from *C*. *sorensianum* until *C*. *anatinum* and then decreased to *C*. *rostratum* (Panel 3 in Fig 5). The age of all examined specimens varied from < 1 year to 12 years, with shell length from 18 to 125 mm. The B/H showed a low variability range with an increasing trend with age (R^2^ = 0.996) (Panel 4 in Fig 5). Individuals from distinct locations showed different growth rates, with the mean growth of individuals from all populations being shown in Panel 5 in Fig 5. Age is more closely connected with the B/H index values (R^2^ = 0.996) than with shell length (R^2^ = 0.928) with its higher accuracy reflecting its logarithmic dependence (R^2^ = 0.964). In addition, B/H values increase with shell length in groups that have been identified as separate species (Fig 6). Shell shape, wing development and shell convexity change with shell length, corresponding to six different size-age group, in the analyzed TCS-CM species (Table 3).

All of the Comparatory species described from the study area correspond to at least another distinct TCS-CM species with a disjunct range (Table 3; S1–S6 Figs). While for *C*. *piscinale* and *C*. *rostratum* only a single additional species can be recovered (Table 3; S3 and S6 Figs), two species for *C*. *sorensianum* and *C*. *nilssonii* (Table 3; S1 and S2 Figs), three for *C*. *ponderosum* and even four additional species can be recovered in the case of *C*. *anatinum* (Table 3; S4 and S5 Figs). Additionally, specimens identified as *C*. *anatinum*, *C*. *piscinale*, and *C*. *ponderosum* can all be recognized as *C*. *convexum* with a total overlap of the curvatures of the AMCVO (Table 3; S3–S5 Figs). Furthermore, specimens described as *C*. *anatinum* can be assigned as *C*. *ponderosum* and vice-versa (Table 3; S4 and S5 Figs).

### Molecular analyses

**Fig 7:**
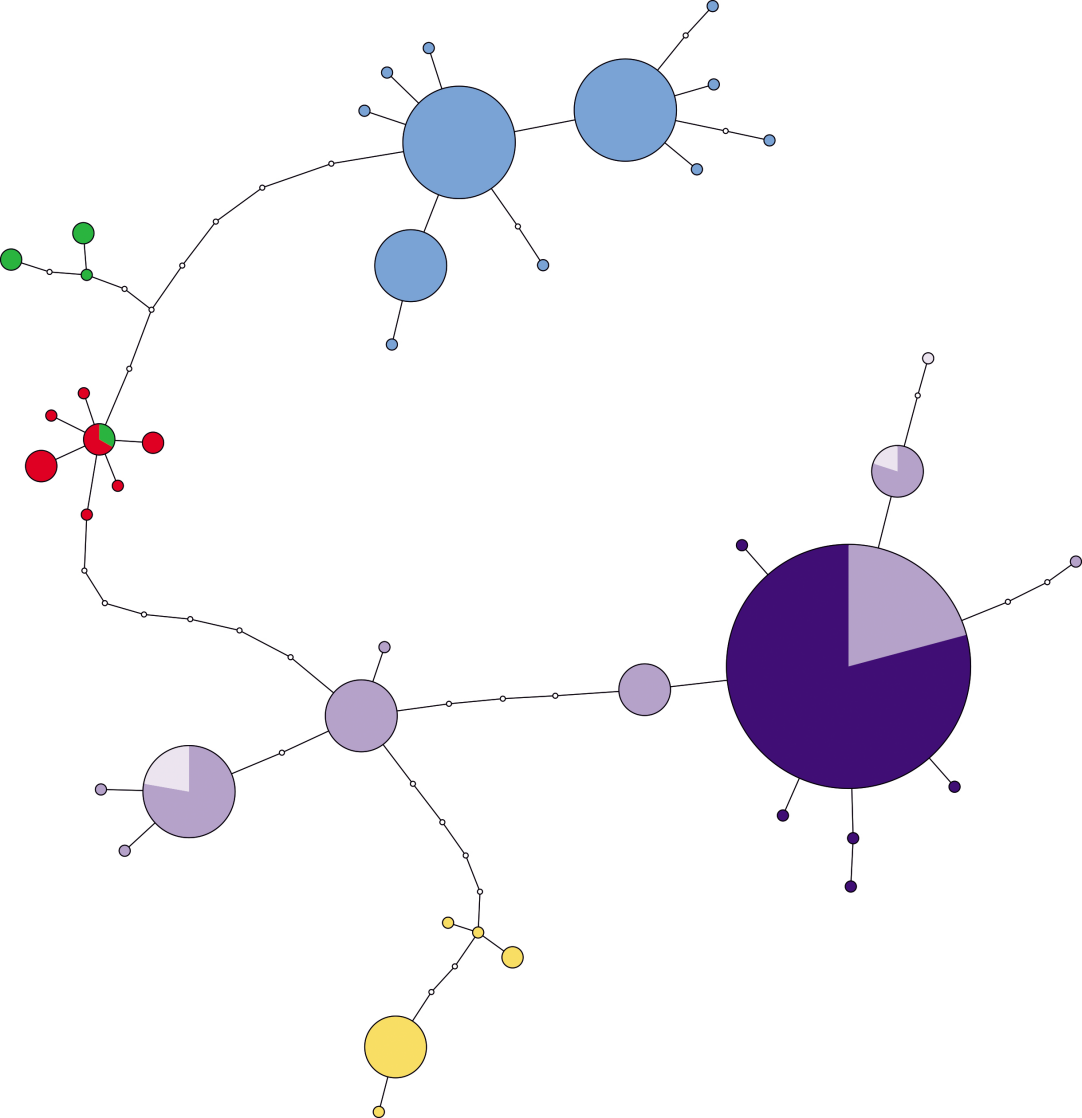
COI haplotype (TCS) network showing the relationships *A*. *anatina* haplotypes. Colour codes correspond to the 5 haplogroups: Blue—Northwest Iberia; Green—Southcentral Iberia; Red Southwest Iberia; Purple—Europe; Yellow—Western Mediterranean. Within the European haplogroup, Russian haplotypes are represented in dark purple and Ukrainian in light purple. Circle size is proportional to the observed haplotype frequencies, white dots represent unobserved haplotypes.

The aligned COI dataset presented a mean length of 578 bp with 21 polymorphic and 14 parsimony informative sets. No indels and no unexpected codons were observed in the corresponding amino-acid translation. TCS produced a single network shown in Fig 7. The 25 newly sequenced individuals from the study area, previously identified as six distinct TCS-CM species, resulted in five haplotypes (Dark Purple: Fig 7) all clustering within the previously recognized European *A*. *anatina* mitochondrial haplogroup (Purple: Fig 7) presenting a low intraspecific genetic diversity (*p*-distance = 0.6%). Eighty per cent of the individuals share a common haplotype with the remaining 20% representing 5 single private haplotypes. Moreover, the common haplotype is also shared with individuals from Poland and Sweden. From the six newly sequenced individuals from Ukraine, we retrieved four haplotypes also clustering within the European haplogroup (Fig 7). Both molecular species delineation methods applied in the COI dataset (Table 2) resulted in the identification of a single MOTU and therefore of a single species.

## Discussion

The present study represents the first comprehensive investigation of the TCS-CM *Colletopterum* species (Anodontinae) from Lake Baikal and adjacent territory of Transbaikalia. The integrative results here presented clearly show that all collected specimens belong to the species *Anodonta anatina*.

Molecular results additionally show that all analyzed specimens cluster within the European *Anodonta anatina* previously described mtDNA clade [25], with most individuals sharing haplotypes with specimens from Europe (Fig 7). Furthermore, no genetic subdivision was detected within the specimens from the studied region, discarding further division in subgenera and species. Both molecular species delimitation methods indicate a single MOTU, corroborating the existence of a single species, i.e. *A*. *anatina*. These results are further confirmed by the morphological and morphometric analyses.

According to Bogatov et al. [16], *Colletopterum* was separated from *Anodonta* by changes in shell size, umbo sculpture and shell surface. In the present study, all specimens were undoubtedly identified as a single species. The shell differences that *A*. *anatina* presents compared to other anodontine species within *Anodonta* are very weak. These slight differences do not support the separation of *A*. *anatina* and other *Anodonta* species (i.e., *Anodonta cygnea* and Western North American *Anodonta*) into distinct genera and should correspond to infrageneric differences. In fact, since anodontine species are known to present a high shell plasticity, these differences were even overlooked by the most comprehensive classification system carried by Haas [11], combining *A*. *anatina* with *A*. *cygnea* forms under a single species. Additionally, *A*. *anatina* clusters with *A*. *cygnea* and *Anodonta nuttalliana* in a monophyletic clade in the most recent molecular phylogeny [23].

According to Bogatov et al. [16], *Colletopterum* was separated into two subgenera based on shell characteristics, i.e presence/absence of a clear wing, but the same author noted that in mature or strongly convex specimens, this wing may be reduced. The present study revealed a considerable variability on the wing expression (S1–S6 Figs), without any correspondence to the two putative *Colletopterum* subgenera. Again, the data here presented discards the existence of a subdivision within the putative *Colletopterum* species, i.e within *Anodonta anatina*.

The main characteristics used to delimit each of the 12 TCS-CM *Colletopterum* species are the arc of maximal convexity of the valve’s outline (AMCVO) and the index of convexity (ratio of shell width to shell height) (Panels 2–4 in Fig 2) [16]. The present study shows that these features are not species specific. In fact, each TCS-CM species might be identified by the AMCVO as two-three, or even four different species (Panel 12—in S3 Fig) from both putative subgenera (S1–S6 Figs). No significant differences were found with regard to double-looped umbonal sculpture, soft part anatomy, and larval and adult shell morphology that supports the distinction of *Colletopterum* in two distinct subgenera (Panels 1–3 in Fig 4) [43].

It is well established that shell shape of unionoids is strongly influenced by both biotic and abiotic factors such as age, size, sex of the mussels, and temperature, hydrological, hydrochemical and trophic conditions [5], [6], [10]. Furthermore, many unionid and especially anodontine mussels, exhibit considerable intraspecific shell shape variation, which is often related with their occurrence in distinct habitats, shifts of metabolism at sexual maturity or even by changes in allometric growth and other physiological characteristics [10], [44], [45], [46], [47]. This influence in shell shape is also shown here for *A*. *anatina*, where B/H values increase with shell length. These values may be divided in six classes (Fig 6), each including 2–3 TCS/CM species that present an overlap of the B/H values. The same classes can also be recovered using the other two morphometric indexes H/L, and l/L values. However, these morphometric classes are composed of TCS-CM species from distant geographic ranges. Since no other independent morphological, anatomical or molecular character exhibit the same pattern, it clearly shows that these indexes are being influenced by environmental factors and should not be used to divide species. Furthermore, all collected young individuals were included in the *C*. *sorensianum* class group which indicates that age is clearly influencing not only the B/H but also the H/L, and l/L values (Figs 5 and 6). In fact, using all specimens across all TCS-CM species, the B/H index show very little variability with age, supporting the idea that shell shape is closely linked with growth (Fig 6). Furthermore, the sex of the animal might also influence shell shape in *Anodonta* [10], and consequently influencing the B/H, H/L, l/L morphometric indexes and AMCVO curves. According to Zieritz & Aldridge [10] *A*. *anatina* is dimorphic, with females being more inflated than males from the same size-age, due to the presence of swollen marsupia filled with glochidia during gravidity.

In summary, morphological, morphometric and molecular genetic data show that all TCS-CM species from both previously recognized subgenera of genus *Colletopterum* represent size-age class groups of one polymorphic species, i.e. *Anodonta anatina*, widespread in the Lake Baikal, Transbaikalia, Siberia and Europe.

## Supporting information

S1 Fig Shell shape and species identification according to TCS-CM of specimens identified as *C*. *sorensianum*. **S1.1** –holotype *C*. *sorensianum* (figure from Starobogatov et al., 2004: 127, Table 26, Fig 5–6), **S1.2** –Lake Baikal, **S1.3 –**Lake Kergendu, **S1.4- S1.6 –**Lake Bol’shoe Eravnoe; **S1.7** –*C*. *subcirculare* from River Don and **S1.8** –*C*. *baeri* from River Volga (figure copy from Bogatov & Kijashko, 2016: Table III, figures 29–30).
(TIF)

S2 Fig Shell shape and species identification according to TCS-CM of specimens identified as *C*. *nilssonii*. **S2.1** –Cherkalov Sor and **S2.2** –Chivyrkuy Bay of Lake Baikal, **S2.3** –Lake Arachley, **S2.4** –Lake Schuchje, **S2.5** –Lake Bol’shoe Eravnoe, **S2.6** –Lake Torma, **S2.7** –Lake Gusinoye, **S2.8** –Lake Kergendu, **S2.11 –**Moscow region (№ 1, ZISP), **S2.9 –***C*. *milaschevichi* from River Volga (holotype № 1, ZISP) and **S2.10 –***C*. *ostiarium* from River Dnieper (№ 7, ZISP) (figures 6.11, 6.9–6.10 reproduced from Bogatov & Kijashko, 2016: Table II-III, fig. 26, 31–32).
(TIF)

S3 Fig Shell shape and species identification according to TCS-CM of specimens identified as *C*. *piscinale*. **S3.1 –**Lake Gusinoye, **S3.2 –**Lake Torma, **S3.3- S3.5** –Lake Bol’shoe Eravnoe, **S3.6** –Lake Kergendu, **S3.7 –**Lake Arachley, **S3.8** –Lake Ivan, **S3.9** –Cherkalov Sor of Lake Baikal, **S3.10 –**River Volga (ZISP, figures reproduced from Bogatov & Kijashko, 2016: Table III, fig. 35: j).
(TIF)

S4 Fig Shell shape and species identification according to TCS-CM of specimens identified as *C*. *anatinum* from different localities. *C*. *anatinum*: **S4.1**- **S4.2 –**Lake Gusynoe, **S4.3 –**Lake Arey, **S4.4** –Lake Schuchje, **S4.5 –**Lake Torma, **S4.6 –**Lake Kergendu, **S4.7** –Lake Bol’shoe Eravnoe, **S4.8** –Lake Ivan, **S4.9** –Lake Arachley, **S4.10 –**Cherkalov Sor, Lake Baikal and **S4.12 –**River Ivitza, Tverskaya region (collection of the Institute of Biology and Soil Science Far East Russian Academy of Sciences, Vladivostok); **S4.11** – *С*. *convexum* from River Don (collection of the Zoological Institute Russian Academy of Sciences, Saint-Petersburg). Figures 8.11–8.12 reproduced from Bogatov & Kijashko, 2016: Table II-III, fig. 27 and 34).
(TIF)

S5 Fig Shell shape and species identification according to TCS-CM of specimens identified as *C*. *ponderosum*. **S5.1- S5.2** –Cherkalov Sor, Lake Baikal, **S5.3** –Lake Schuchje, **S5.4** –Lake Torma, **S5.5** –Lake Gusinoye, **S5.6**, **S5.12 –**Lake Bol’shoe Eravnoe, **S5.7**, **S5.9**, **S5.11** –Chyvyrkuy Bay of Lake Baikal, **S5.8**, **S5.10 –**Lake Arachley, **S5.13** –Lake Kergendu, **S5.14 –**Lake Tasey, **S5.15** –Lake Uzminskoye Lake, European Russia (№ 36, ZISP); **S5.16** –*C*. *apollonicum* from Lake Appolonya, Southern Europe (№ 1, ZISP). Figures 9.15–9.16 reproduced from Bogatov & Kijashko, 2016: Table II-III, fig. 33 and 28.
(TIF)

S6 Fig Shell shape and species identification according to TCM-CS of specimens identified as *C*. *rostratum*. **S6.1 –**Lake Baikal, **S6.2** –Lake Gusinoye, **S6.3** –Lake Kenon, **S6.4** –Germany (№ 1, ZISP), **S6.5 –**River Ivitza, European Russia. Figures **S6.4** and **S6.5** reproduced from Bogatov & Kijashko, 2016: Table III, fig. 36).
(TIF)
