# Supplementary figures and images for: Morphological and molecular analyses of Anodontinae species (Bivalvia, Unionidae) of Lake Baikal and Transbaikalia
Source: PLoS One. 2018 Apr 9;13(4):e0194944. doi: 10.1371/journal.pone.0194944 (PMC5890983; doi:10.1371/journal.pone.0194944)

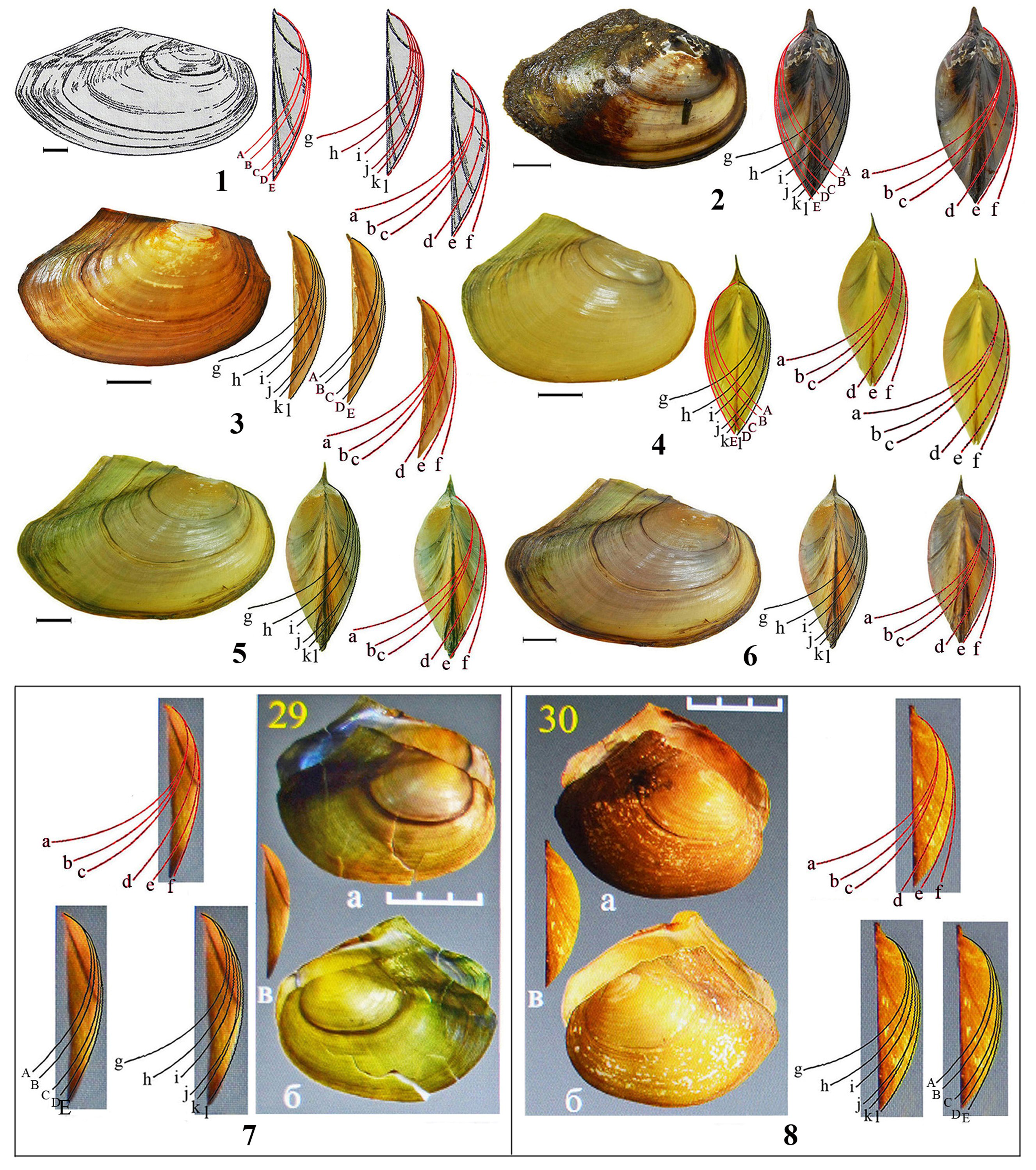

Supplement: S1 Fig — S1.1 –holotype C. sorensianum (figure from Starobogatov et al., 2004: 127, Table 26, Fig 5–6), S1.2 –Lake Baikal, S1.3 –Lake Kergendu, S1.4- S1.6 –Lake Bol’shoe Eravnoe; S1.7 –C. subcirculare from River Don and S1.8 –C. baeri from River Volga (figure copy from Bogatov & Kijashko, 2016: Table III, figures 29–30). (TIF) [file pone.0194944.s001.tif]

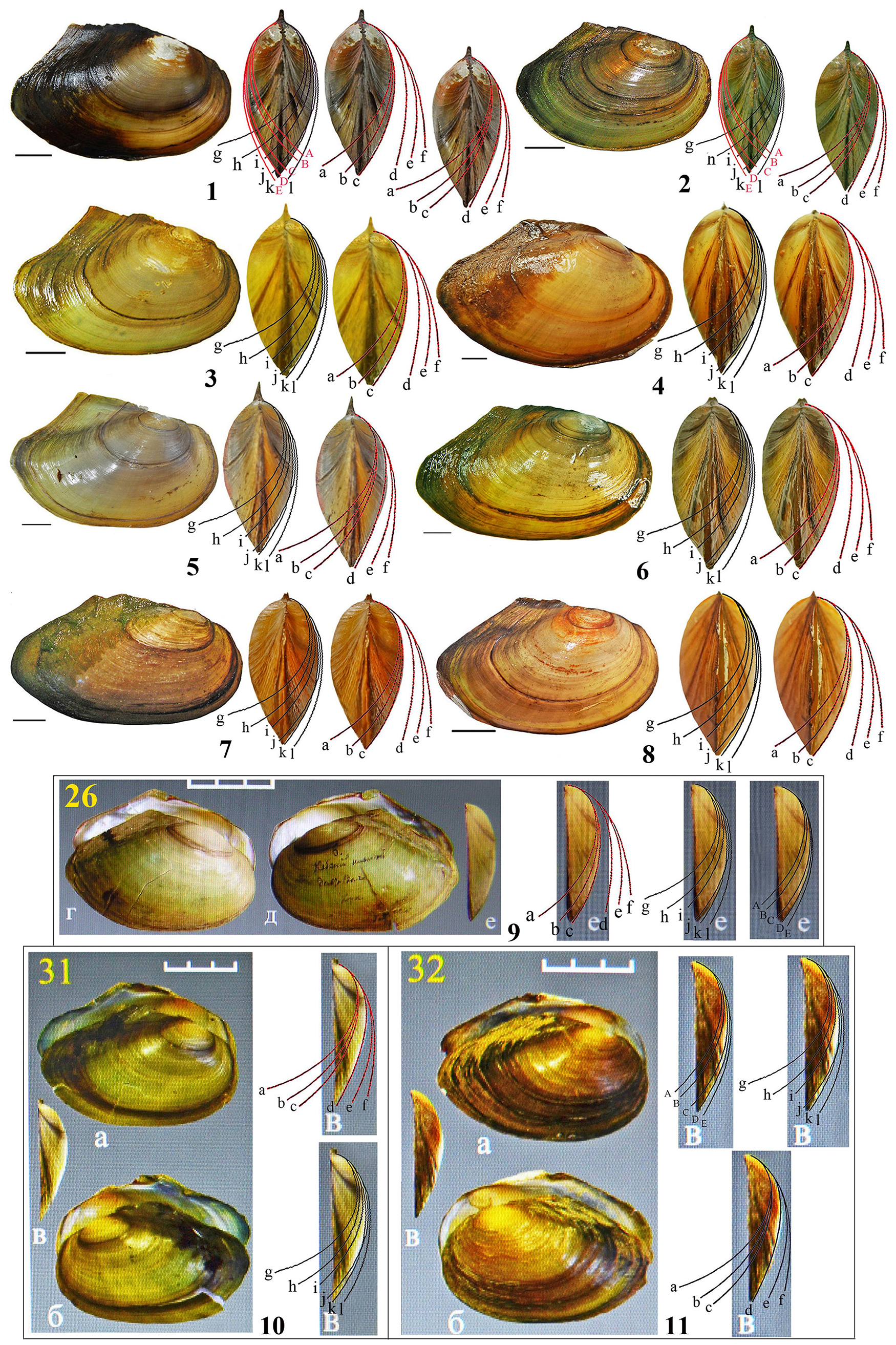

Supplement: S2 Fig — S2.1 –Cherkalov Sor and S2.2 –Chivyrkuy Bay of Lake Baikal, S2.3 –Lake Arachley, S2.4 –Lake Schuchje, S2.5 –Lake Bol’shoe Eravnoe, S2.6 –Lake Torma, S2.7 –Lake Gusinoye, S2.8 –Lake Kergendu, S2.11 –Moscow region (№ 1, ZISP), S2.9 –C. milaschevichi from River Volga (holotype № 1, ZISP) and S2.10 –C. ostiarium from River Dnieper (№ 7, ZISP) (figures 6.11, 6.9–6.10 reproduced from Bogatov & Kijashko, 2016: Table II-III, fig. 26, 31–32). (TIF) [file pone.0194944.s002.tif]

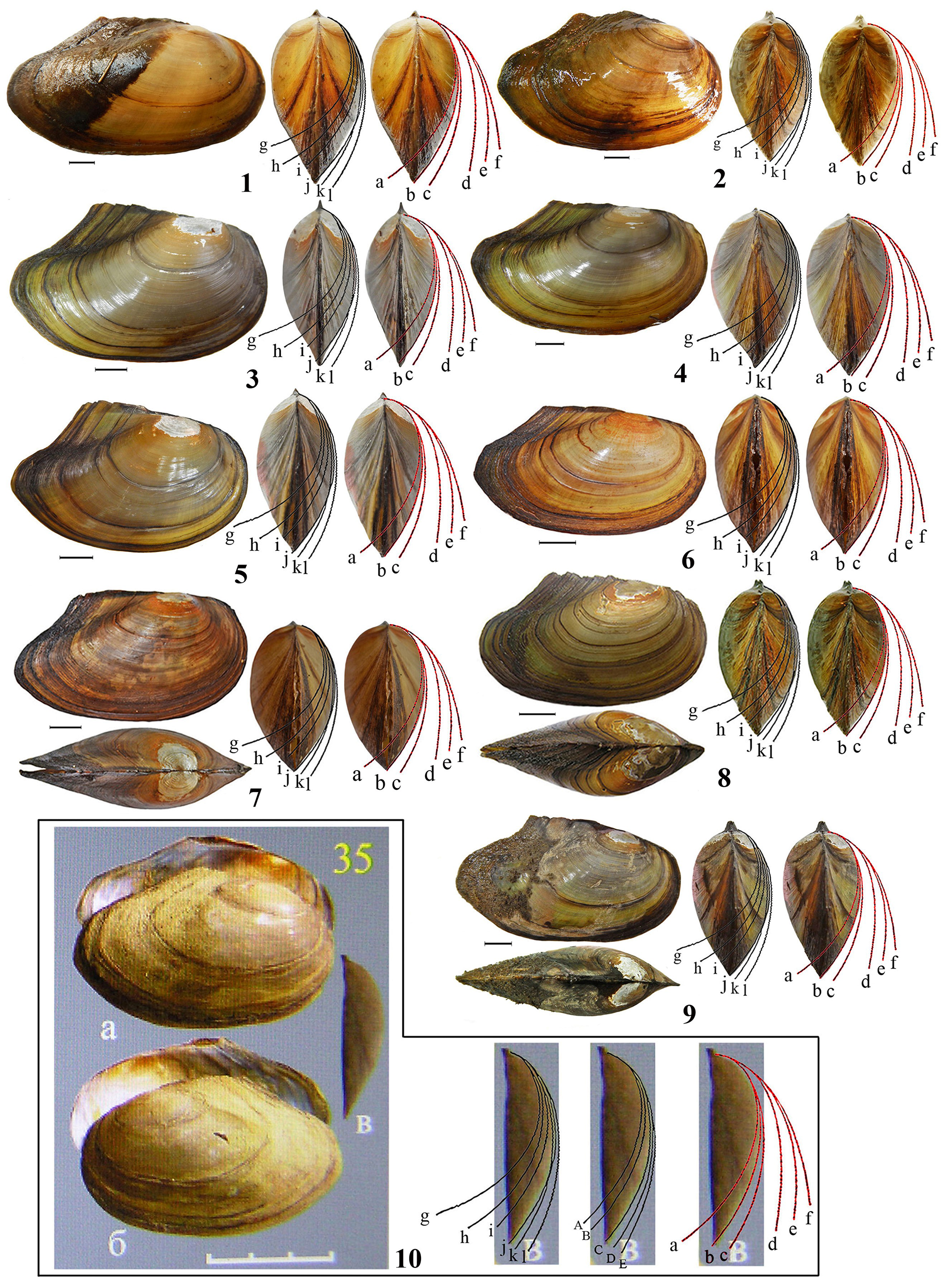

Supplement: S3 Fig — S3.1 –Lake Gusinoye, S3.2 –Lake Torma, S3.3- S3.5 –Lake Bol’shoe Eravnoe, S3.6 –Lake Kergendu, S3.7 –Lake Arachley, S3.8 –Lake Ivan, S3.9 –Cherkalov Sor of Lake Baikal, S3.10 –River Volga (ZISP, figures reproduced from Bogatov & Kijashko, 2016: Table III, fig. 35: j). (TIF) [file pone.0194944.s003.tif]

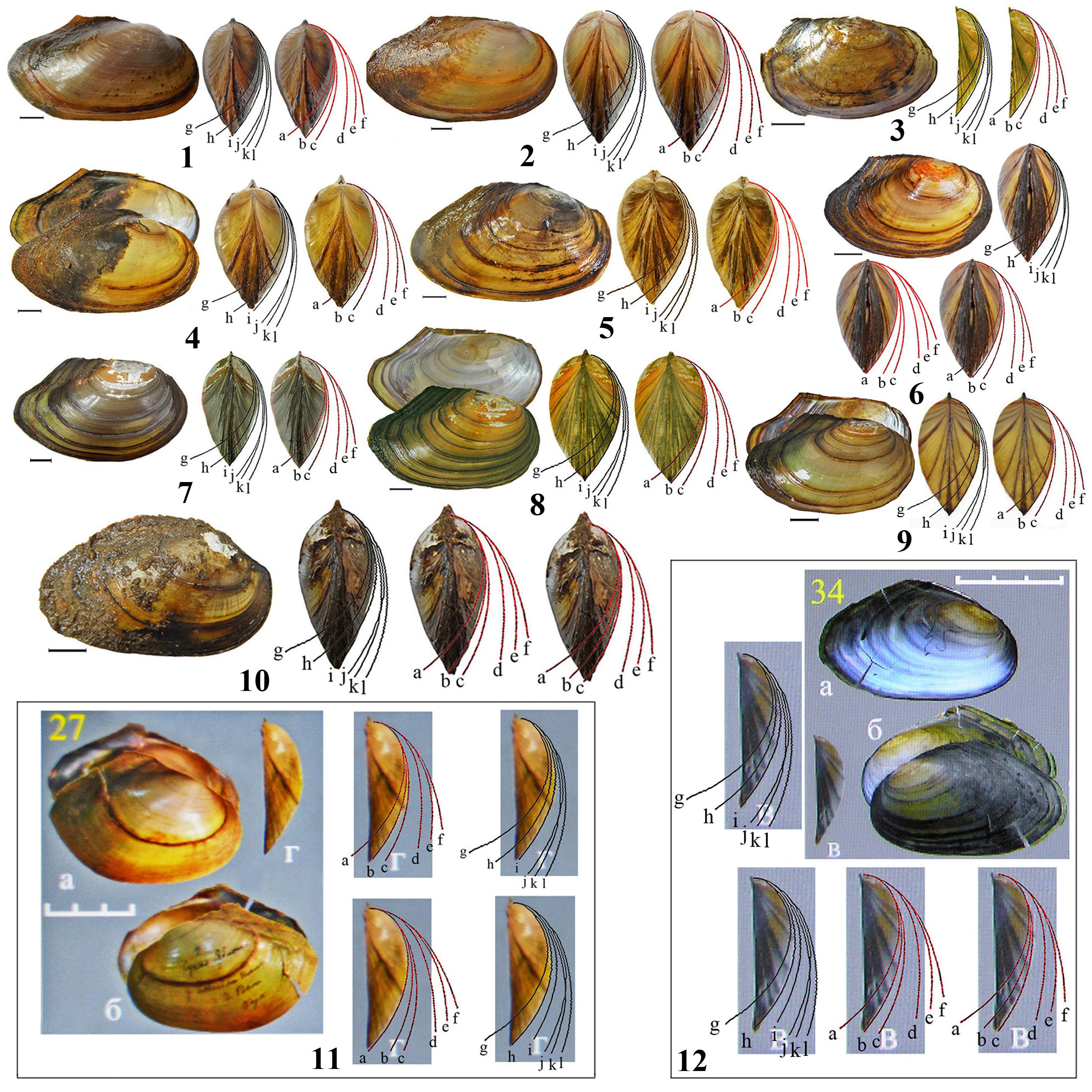

Supplement: S4 Fig — C. anatinum: S4.1- S4.2 –Lake Gusynoe, S4.3 –Lake Arey, S4.4 –Lake Schuchje, S4.5 –Lake Torma, S4.6 –Lake Kergendu, S4.7 –Lake Bol’shoe Eravnoe, S4.8 –Lake Ivan, S4.9 –Lake Arachley, S4.10 –Cherkalov Sor, Lake Baikal and S4.12 –River Ivitza, Tverskaya region (collection of the Institute of Biology and Soil Science Far East Russian Academy of Sciences, Vladivostok); S4.11 – С. convexum from River Don (collection of the Zoological Institute Russian Academy of Sciences, Saint-Petersburg). Figures 8.11–8.12 reproduced from Bogatov & Kijashko, 2016: Table II-III, fig. 27 and 34). (TIF) [file pone.0194944.s004.tif]

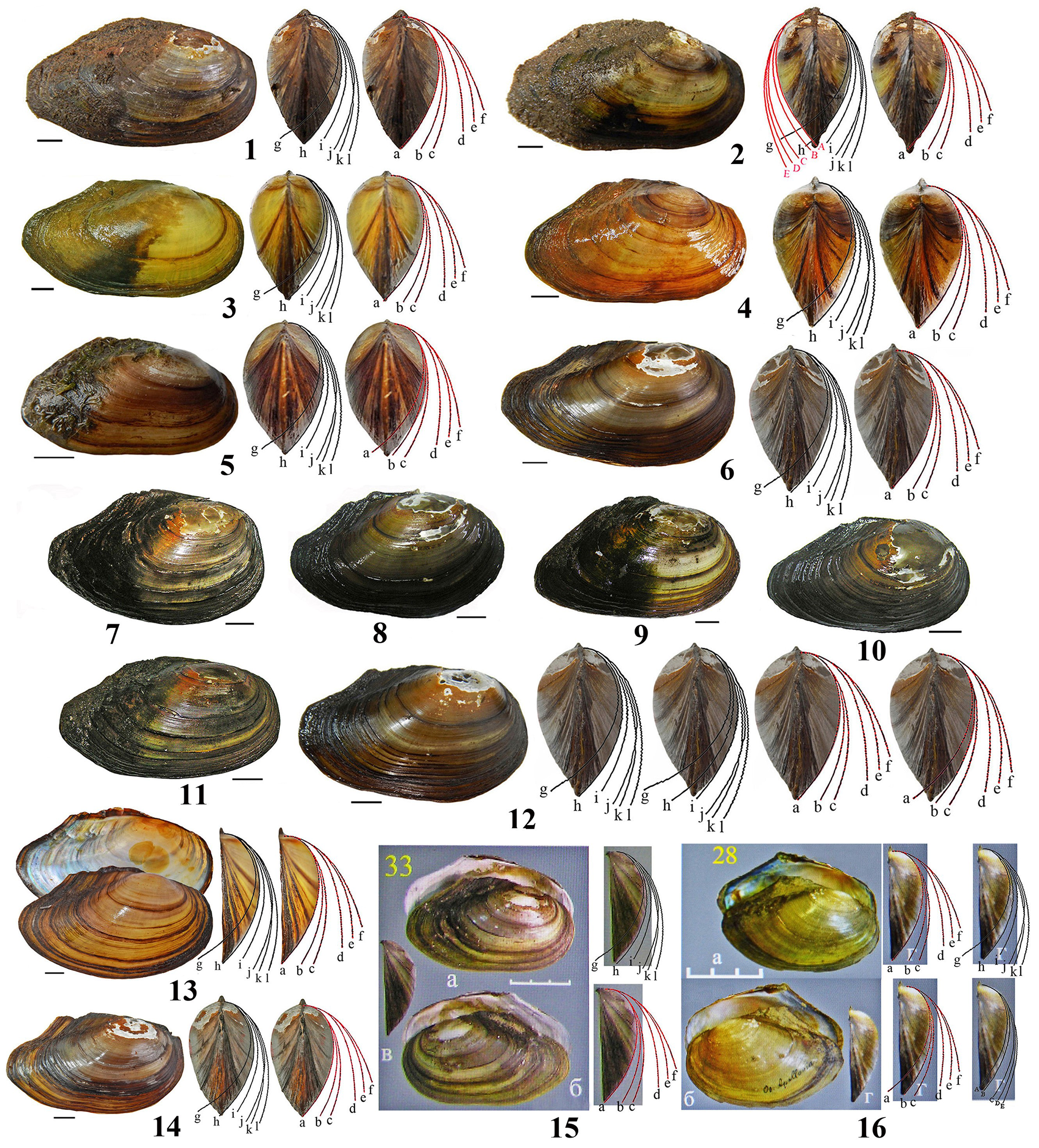

Supplement: S5 Fig — S5.1- S5.2 –Cherkalov Sor, Lake Baikal, S5.3 –Lake Schuchje, S5.4 –Lake Torma, S5.5 –Lake Gusinoye, S5.6, S5.12 –Lake Bol’shoe Eravnoe, S5.7, S5.9, S5.11 –Chyvyrkuy Bay of Lake Baikal, S5.8, S5.10 –Lake Arachley, S5.13 –Lake Kergendu, S5.14 –Lake Tasey, S5.15 –Lake Uzminskoye Lake, European Russia (№ 36, ZISP); S5.16 –C. apollonicum from Lake Appolonya, Southern Europe (№ 1, ZISP). Figures 9.15–9.16 reproduced from Bogatov & Kijashko, 2016: Table II-III, fig. 33 and 28. (TIF) [file pone.0194944.s005.tif]

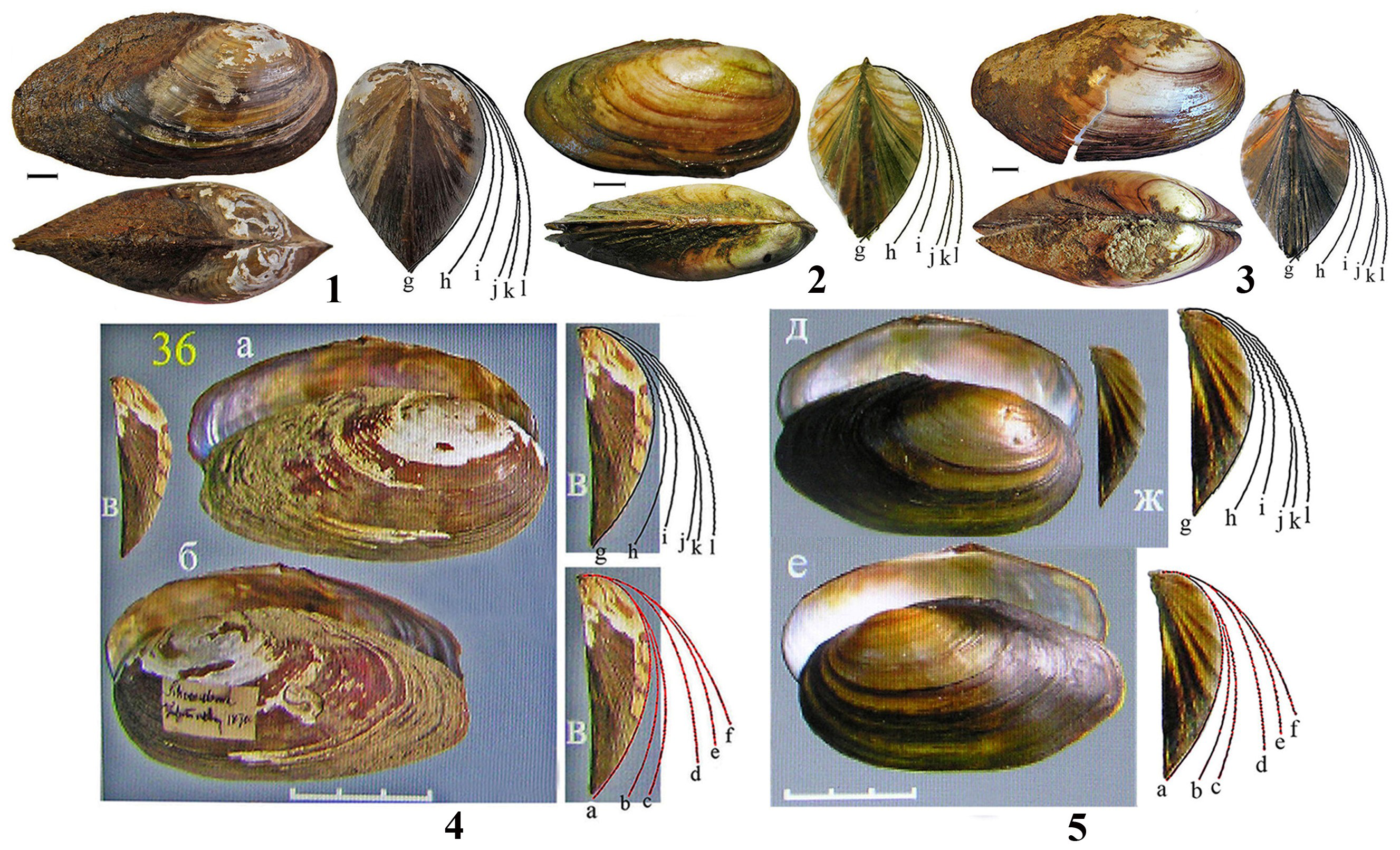

Supplement: S6 Fig — S6.1 –Lake Baikal, S6.2 –Lake Gusinoye, S6.3 –Lake Kenon, S6.4 –Germany (№ 1, ZISP), S6.5 –River Ivitza, European Russia. Figures S6.4 and S6.5 reproduced from Bogatov & Kijashko, 2016: Table III, fig. 36). (TIF) [file pone.0194944.s006.tif]
